# Prosthetic Applications of Short Dental Implants in Limited Bone Height Cases: A Review Article

**DOI:** 10.7759/cureus.73551

**Published:** 2024-11-12

**Authors:** Fatemah B Ibrahem, Mostafa I Fayad, ِAbdel Naser M Emam, Mohamed A Helal, Ibrahim A Abd-Elrahman, Mohammad A Alqhtani, Mohamad A Quassem

**Affiliations:** 1 Prosthetic Dental Science Department, Faculty of Dentistry, Najran University, Najran, SAU; 2 Substitutive Dental Sciences, Faculty of Dentistry, Taibah University, Al-Madina Al-Munawara, SAU; 3 Department of Prosthodontics, Faculty of Dentistry, Al-Azhar University, Nasr City, EGY; 4 Prosthodontic Department, Ministry of Health (MOH), El-Mansoura, EGY; 5 Prosthodontic Department, Faculty of Dental Medicine, Al-Azhar University, Boys Branch, Cairo, EGY

**Keywords:** implant design, implant prostheses, implant size, implant supported prosthesis, mini implant

## Abstract

The decision to rehabilitate insufficient alveolar bone height with short implants is considered an effective treatment. Complicated surgical procedures such as ridge augmentation, vestibuloplasty, and sinus lifting are usually accompanied by high risks such as membrane injury, hemorrhage, nerve affection, and increased time needed for implant treatment with unpredictable healing time. Short dental implants allow for faster treatment and decrease the need for complicated surgeries that are usually associated with standard dental implants, as in all four and all six concepts. A wide variety of short implants for replacing single, multiple, or even prosthetic rehabilitation of completely edentulous patients, either by using hybrid or overdenture prostheses, were documented by several studies. Further clinical investigations are needed to evaluate the improvement of short implant survival rate, the possibility of using short implants for patients with normal ridge height and contour, and also for patients who need maxillofacial prostheses.

## Introduction and background

Many patients suffer from complete edentulism with variant percentages and causes; however, with the recent innovations and progression in dentistry, these situations have been improved. Complete edentulism without rehabilitation with complete dentures causes excessive damage to oral and health conditions [1,2]. Restoration of extremely atrophied ridges with dental implants is still a surgical and prosthetic challenge for dentists and patients [3].

Implant insertion needs enough bone height and width to allow a correct implant placement. In cases of reduced bone height, after advanced reconstructive surgical treatments, a standard implant can be inserted, which results in prolonged, expensive treatment and more postoperative complications for the patient [4,5].

Patients are usually satisfied with implant treatment with minimally invasive strategies, especially if they are suffering from relatively implant contraindications such as controlled diabetes or transient systemic complications. For such patients, placement of short implants with minimally invasive surgery may be more suitable and convenient for prosthetic rehabilitation [6].

For many years, authors documented that implant survival rates increased by inserting the longest implant, as short implants showed high failure rates. However, with the modern application of the concept of “stress-minimizing surgery,” short implants have gained more popularity [7].

Literature review process

A systematic search strategy was designed to conduct the literature review. The primary databases searched included PubMed/Medline, Scopus, Web of Science, Embase, and the Cochrane Library, with Google Scholar serving as an additional resource for broader coverage.

Keywords employed in the search included “short dental implants,” “limited bone height,” and “prosthetic applications,” supplemented by terms such as “reduced alveolar bone,” “implant-supported prostheses,” and “bone augmentation alternatives.” Boolean operators (AND, OR) were used to combine keywords effectively for targeted results.

To ensure relevance, filters were set to include peer-reviewed studies in English. The study types included clinical trials, systematic reviews, case studies, and cohort studies, ensuring for a targeted search in PubMed, specific MeSH (Medical Subject Headings) terms were utilized to refine results related to short dental implants in cases of limited bone height. Primary MeSH terms included “Dental Implants,” “Bone Resorption,” and “Prostheses and Implants.” Secondary MeSH terms were “Alveolar Ridge Augmentation,” “Bone Density,” and “Dental Prosthesis Design,” which were relevant for highlighting studies focusing on prosthetic applications under challenging bone conditions. Additionally, terms such as “Immediate Dental Implant Loading” and “Dental Prosthesis, Implant-Supported” were included to capture variations in implant placement and loading protocols that influence prosthetic outcomes.

The inclusion criteria for this review were tailored to ensure that only the most relevant studies were considered. Included studies had to focus on the use of short dental implants for prosthetic applications in cases with limited bone height, offering clinical insights and outcomes. Priority was given to clinical trials, systematic reviews, and case reports that provided quantitative or qualitative data. The research had to be published in peer-reviewed journals to ensure credibility. Additionally, studies detailing prosthetic stability and patient satisfaction as outcomes were given preference.

Exclusion criteria were established to maintain the review's focus and quality. Studies that focused exclusively on traditional-length dental implants or unrelated prosthetic treatments were excluded. Non-peer-reviewed publications, opinion pieces, and conference abstracts were disregarded due to their limited reliability.

**Figure 1:**
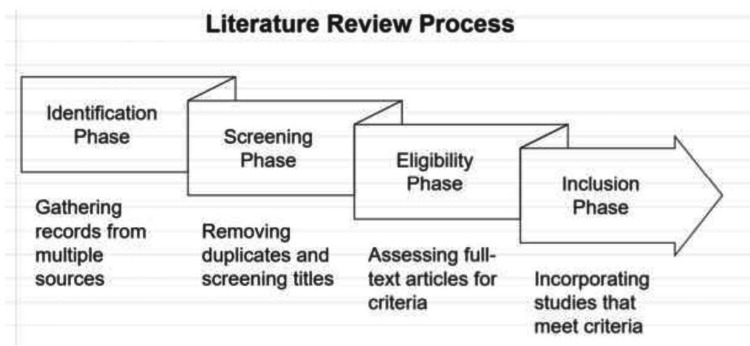
Literature review process

For clarity, the review followed a structured process represented in a flow diagram. The identification phase involved gathering records from multiple databases and additional sources. In the screening phase, duplicate records were removed, and titles and abstracts were screened for relevance. During the eligibility phase, full-text articles were assessed, and those not meeting the criteria were excluded. Finally, the inclusion phase comprised studies that matched the specified criteria and were incorporated into the final review. This systematic approach ensured a thorough assessment of the literature, enhancing the review's robustness (Figure 1).

## Review

Short implant

A short implant was defined as an implant that has a length less than 10 mm (recently, it may be implants shorter than 8, 7, or even 6 mm); it was proposed to be easier and more effective for the placement in severely absorbed ridges and at areas of vital structure approximation [8,9].

The Food and Drug Administration (FDA) gave the acceptance to the Bicon system in 2008 to launch a 5-mm-height implant. Also, Straumann produced a 4-mm-high (Roxolid) implant that showed survival rates of 94% after a five-year follow-up [10].

Advantages of using short implants

The placement of short implants in treating edentulous patients eliminates the need for complicated surgeries to place conventional implants. Patient satisfaction will be increased as it avoids augmentation procedures, which result in short, inexpensive treatment options [11,12]. Applying short implants in extremely resorbed mandibles (less than 10 mm bone height) might reduce the risk of mandibular fracture during penetration in the symphysis region, preserving the integrity of the lower mandibular cortex [13,14].

Implant insertion is easier, and the incidence of bone overheating is reduced as osteotomy preparation is simple and shorter, which allows better irrigation entry during drilling [15].

Factors affecting the success of short implants

Several studies concluded that many factors directly influence the success rates of short implants: implant diameter, length, number, stress distribution around the implant, marginal bone loss, implant thread design, and loading strategy for the prosthesis.

Implant diameter and length

Implant length or geometry had a lower effect on decreasing stresses than diameter. Increasing the diameter resulted in reducing bone stresses by up to 30%, while increasing length caused stress reduction by up to 8% [16]. Increasing implant diameter resulted in better stress distribution within the bone around the implant that may resulted from the osseointegrated with the surrounding cortical plates and increased surface area of contact [17].

Finite element analysis supported the concept that increasing implant diameter could increase the implant bone surface area and allow more occlusal force distribution with more implant stability. It also concluded that the length of the implant may not affect the transfer of occlusal stresses to the bone around the implants [18,19].

Implant number

Many studies have expected that increasing the number of implants would result in a decrease in the stress values in the bone surrounding the dental implant. Also, stress is distributed over wide surface areas [20,21].

Tabrizi et al. demonstrated that increasing the number of short implants results in a decreased percentage of marginal bone loss compared to using fewer short implants in rehabilitating the mandibular posterior fixed prostheses [22].

Meijer et al. showed that stress distribution was unequal between two and four implant models in severely resorbed mandibles. Also, increasing implant numbers didn’t lead to a reduction in stress. The highest stress values were measured in two implant-retained overdenture models, and the stress distribution in three and four implant-supported models was found to be similar [23].

Some concerns have been raised regarding the application of three implants to support overdentures because the bone around the middle implant may be associated with higher stress, particularly when a load is applied to posterior teeth [24].

Stress distribution

Pierrisnard et al., using a finite element analysis, showed that the stresses to the implant were greater with increased implant length; also, the abutment screw showed the same results. Longer implants revealed more stress than short implants [25].

Comparing short implants with diameters of 3.75 mm and 5 mm showed that oblique loading results in more stress in the cortical bone while axial loading reveals less stress. The results showed better stress distribution in the bone with increasing implant diameter regardless of the connection type [26].

Memari et al. evaluated the amount of stress distributed in the crestal bone around the short and long implants and concluded that there was no difference between them after loading the prostheses. Short implants showed less but non-significant stress values compared to long implants. Short implants would be a treatment option for implant-supported prostheses and lower jaw overdentures [27].

Marginal bone loss

Albrektsson et al. stated that if the annual bone loss is less than 0.2 mm after one year of implant loading, it could be considered a successful implant. Also, Adell et al. (29) revealed that bone loss of 1.5 mm after one year and then 0.1 mm annually was a criterion for implant success [28].

Marginal bone loss (MBL) for short implants was significantly lower than for conventional implants after three years of functioning. The difference may be due to conventional implants' lower diameters than short ones. It was suggested that there is no significant correlation between short implants' crown-to-implant ratio (CI) and MBL in the posterior areas. The MBL was passively affected by the usage of prostheses with cantilevers [29,30].

Monje et al. concluded that a non-significant regression slope was found between MBL and implant length using a random effect model. Therefore, there is no influence between MBL around implants and implant length [31].

Implant design

The implant-bone surface area can be increased by increasing the thread numbers and using a deeper thread design [32]. The square thread design showed a greater amount of bone-implant contact compared to the V-shape and reversed buttress thread designs. Squared and buttress threads dissipated the axial forces mainly through compressive force, while V-shaped and reversed buttress-threaded implants transmit axial force through a combination of compressive, tensile, and shear forces with shear force 10 times greater [33,34].

Short implants with double thread design showed a higher success rate in addition to higher primary stability in D3 and D4 bone [35].

Rough microtopography of the implant surface increases the bone-implant contact surface area and enhances osseointegration compared to a smooth surface [36,37]. It has been suggested that the design and implant body material could reduce the risk of failure and have more effect on stress distribution than the difference in length. Also, the extra-short implants showed some complications, such as implant body fracture, and loosening of the screw increased with the increase in stress concentration [38].

Loading strategies

Han J et al. found that the conventional loading protocol of short dental implants is much safer than early loading, especially in cases with low bone quality, systemic diseases that affect vascularity or cause delayed wound healing, and severe periodontal diseases. It was suggested that regarding the implant success rates, the immediate loading protocol for inserting short implants might be safe, with no significant difference between immediate and conventional loading protocols (Table 1) [39,40].

**Table 1:** Summary of Key Factors Influencing the Success of Short Dental Implants

Factors	Description and impaction
Implant diameter and length	Increasing implant diameter improves stress distribution by osseointegrating with surrounding cortical plates and increasing the surface area of contact [17].
Implant number	Increasing the number of implants helps decrease stress on surrounding bone, improving stability and reducing risks [21].
Stress distribution	the amount of stress distributed in the crestal bone around the short and long implants was evaluated and there was no difference between them after loading the prostheses [27].
Marginal bone loss	Preservation of marginal bone around the implant neck is crucial. Marginal bone loss (MBL) for short implants was significantly lower than for conventional implants after 3 years of functioning [30].
Implant design	Increasing thread numbers and using a deeper thread design enhances the implant-bone contact area, leading to better osseointegration [32].
Loading strategies	Conventional loading protocols are safer, especially in low bone quality cases, compared to early loading strategies [39,40] .

Cannizzaro et al. illustrated after nine years of follow-up that immediate or early loading of short implants placed using a flapless method could be considered a long-term clinical success as implants placed in both upper and lower jaws with immediate loading [41].

Rosa et al. demonstrated that an all-on-four prosthetic protocol with immediate loading on a short implant was successful in extremely resorbed mandibular ridges after a four-year follow-up [42].

Prosthetic application of short dental implants

Short Implant-Supported Overdenture

The placement of two short implants with a 4.1mm diameter loaded with locator attachments to support unpleasant conventional dentures showed high survival rates of 94.7% and marginal bone loss within the acceptable limits (.17mm per year) [43].

Maryod et al. demonstrated that finite element analysis of poly-ether-ether-ketone (PEEK) and polymethylmethacrylate (PMMA) overdentures material using four short implants and locator attachments under the proposed load results in no failure in any component of the model; stress values were found within the acceptable physiological limits. That may be due to using four implants to support the overdenture, which distributes the load more evenly. Also, the use of resilient, low-profile locator attachment. PMMA overdenture deformed slightly smaller than PEEK one. The flexible caps and short implants in the case of PEEK overdentures showed very low deformation and stress values compared to PMMA ones under oblique loading [44].

Stress distribution levels in three and four short implant-supported models were found to be similar. Increasing the implant numbers provides more stress distribution on the side surfaces of the implants and decreases compressive stress on the cortical bone surrounding the implants. Regardless of transverse mandibular width, three short implant-supported overdentures should be recommended to treat extremely resorbed edentulous mandibles. Two short-implant retained overdentures could be used as a prosthetic option for extremely resorbed lower ridges, and photo-biomodulation significantly affects the healing and osseointegration of dental implants [45].

Using four short implants with an implant-retained overdenture is one of the options for prosthetic restoration of the severely resorbed lower arch that resulted in minimal marginal bone loss and a survival rate of up to 88%. Also, the tissues surrounding the short implants were healthy during the follow-up period [46].

Short Implant Support Hybrid Prosthesis

Falisi et al. placed six short implants to support a metal-resin prosthesis and provided more implant surface area supporting the prosthesis. After a one-year follow-up, this treatment may be affordable for the patient and have the advantage of predicting an excellent, long-lasting clinical outcome. Also, radiological evaluation showed healthy bone around the implants with osseointegration signs. The prosthesis was found stable, and patient satisfaction was high [47].

A comparison between a full-arch hybrid prosthesis with distal cantilevers supported by five short implants positioned in the non-atrophic mandibular anterior area and another supported by five conventional implants showed that after a three-year recall, none of the implants had been lost. Short implants could be used in the reconstruction of a total edentulous lower jaw, even in the non‐atrophic cases. Rehabilitation of the atrophied mandible and maxilla using a short implant supported by a screw-retained metal resin prosthesis showed no complications and increased patient satisfaction with both function and aesthetics five months after initial loading [48].

Short Implant Support Fixed Prosthesis

It was found that 6-mm-long implants supporting single crowns loaded within seven weeks result in minimal marginal bone loss similar to that of 10-mm-long implants during five years. The implant loss with signs of crown implant mobility was higher in short implants, which may result from fracturing the bone surrounding the implant rather than excessive marginal bone loss [49].

The early loading of single crowns supported by 6mm moderately rough surface short implants demonstrated a marginal bone loss of 0.7 mm after follow-up for five years [50].

The short dental implants in the upper showed higher marginal bone loss than in the lower, with a survival rate of 93.3%. Short implants may be used as a line of treatment to support fixed partial prostheses in both the upper and the lower, with caution to their effect on marginal bone loss [51].

Reconstruction of the edentulous atrophic mandible with a metal-free fixed prosthesis supported by four short implants showed no significant bone loss and an implant rate success of 95% [52].

Four short implants were bilaterally placed in the mandibular edentulous jaw in the area of the first molar and canine to support a fiber-reinforced composite (FRC) substructure of a prosthetic framework. FRC supplied in milling blocks for the CAD/CAM technique. The main advantages of this protocol were minimally invasive procedures, excellent aesthetics and performance, and lower cost [53].

Fixed dental prosthesis (FDP) that replaced the most distal standard implants with short implants either placed tilted or not to support a distal cantilever (10 mm) reduced the destructive forces on implants supporting an FDP with no mechanical loading difference between them compared to that with a distal cantilever [54].

Short Implants Supporting Removable Partial Dentures

Insertion of one short implant in the distal extension areas to support Kennedy class I and class II RPDs showed high survival rates with minimal prosthetic complications, resulting in improved patients' oral health [55].

The placement of two short implants with different lengths and diameters in the premolar and the molar regions on each mandibular distal extension of a Kennedy class I RPD showed a significant increase in masticatory efficiency during recalls. The position of short implants had no significant effect. The clinical and radiographical evaluation of implants, teeth, and RPDs was acceptable, with no complications [56].

A 3D finite element model of a distal extension RPD supported with 7- and 10-mm implants and angles of 0°, 10°, and 15° revealed that changing the length of the implant had little effect on the increase of stress concentration in the implant and decreased of stress in the periodontal ligaments (PDL). While increasing the inclination of the implant significantly increased the implant stress concentration and decreased stress in the PDL [57].

The survival rates of short implants

Short implant placement in mandibular ridges revealed an increased success rate (92.89%) more than maxillary inserted implants (87.83%); the maxillary placement of short implants presented a challenge for implant success [58].

The survival rate of short implants was less than conventional implants after three months. Short implants would offer a suitable option for the reconstruction of atrophied mandibular ridges, reducing the need for complicated bone surgery to insert a proper implant. Patients should be informed about the lower survival rate of the short implant compared with conventional implants before implant insertion to get rid of patients who are over-expectations [59].

The short implants were found to have lower survival rates and higher risks of failure than longer implants (>6 mm) during five years of recall. Also, lower prosthesis survival rates over short implants were documented than those over longer implants [60].

Short implant versus bone augmentation

Dias et al. stated that after one year of functional loading, the survival of the implants was higher for short implants than for long implants inserted after ridge augmentation surgeries. Short implants are documented with high success rates compared to complex surgical interventions in the treatment of highly resorbed ridges. Older studies showed higher failure rates of the machined-surface implants placed in areas with poor-quality bone [61].

It was suggested that a short implant provide low-cost, acceptable, time-saving treatment instead of a long implant placed in the augmented bone while rehabilitating a completely edentulous atrophic maxilla. Significant failures occur with long implants in different augmented sites. Short dental implants were responsible for one-third of the technical complications, while a sinus lifting surgery would have a one hundred percent rise in surgical complications. Also, conventional dental implants had a triple increase in the chance of complications during insertion compared to short implants. A 5mm × 5mm implant gives the same results as a standard long implant placed in augmented bone after three years of loading. The short implant may be the first choice compared to bone augmentation [62].

## Conclusions

The short implant placement reduces the need for difficult bone reconstruction surgeries. Also, the morbidity of the patients, as well as the cost and treatment time, were reduced. Caution should be taken while choosing implant diameter and loading protocol. The short implant provides lines of treatment for severely resorbed jaws similar to standard-length implants (Implant-supported overdentures, hybrid prosthesis, or fixed prosthesis). Further clinical investigations are needed to illustrate the effect of short implants on the prosthetic function, such as masticatory efficiency, retention, stability, and patient satisfaction.
